# Vacuum Deposition of Triple-Halide Wide-Bandgap Perovskites Enabled by Sublimation of Mixed Organic-Halide Pellets

**DOI:** 10.1021/acsmaterialslett.5c01161

**Published:** 2025-10-16

**Authors:** Manuel Piot, Lidón Gil-Escrig, Federico Ventosinos, Cristina Roldán-Carmona, Anna Robinson, Javier A. Schmidt, Michele Sessolo, Henk J. Bolink

**Affiliations:** † Instituto de Ciencia Molecular, 16781 Universidad de Valencia, Paterna 46980, Spain; ‡ Instituto de Física del Litoral (IFIS-Litoral), 428228 CONICET-UNL, Santa Fe S3000GLN, Argentina; § 417037 Oxford Photovoltaics Ltd, Yarnton OX5 1QU, United Kingdom

## Abstract

Controlling the sublimation of organic compounds during perovskite deposition via coevaporation is challenging. The sublimation behavior of these materials depends strongly on their purity, and their low sticking coefficient on sensors complicates deposition rate monitoring, hindering reproducibility, particularly when multiple organic sources are involved. We introduce a novel approach in which the precursor powder is pressed into a pellet, reducing material consumption and pressure fluctuation during coevaporation. This pellet can also incorporate multiple compounds, enabling, for example, the simultaneous sublimation of methyl­ammonium iodide and chloride from a single source. Combined with a Pb­(I_1–*x*_Br_*x*_)_2_ source, this method makes it possible to deposit triple-halide MAPIBrCl perovskite films. We confirm the incorporation of chloride in the perovskite lattice, which proves to be beneficial for the charge transport properties of the film, increasing the fill factor in wide-bandgap solar cells. Using this approach, we achieved a champion PCE of 19.5% for a 1.66 eV bandgap perovskite.

## Graphical abstract

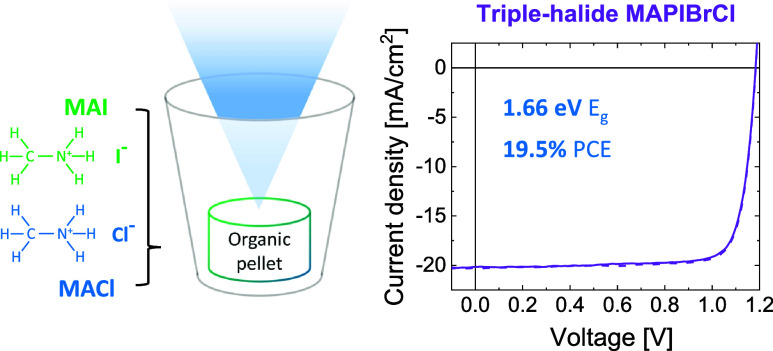

Perovskite solar cells (PSCs) have rapidly become very promising candidates for the next generation of photovoltaics. Metal-halide perovskites combine a variety of optoelectronic properties particularly suitable for this application, such as high absorption coefficient due to a direct bandgap, high charge carrier lifetime, and tunable bandgap.^1–3^ In addition, perovskites are cost-effective and easily prepared at the lab scale. As such, these materials have been the focus of intensive research over the past 15 years, resulting in a substantial and rapid development that brought the state-of-the-art power conversion efficiency (PCE) to 27.0%.^4^ Although perovskite deposition via solution-processing received comparatively more attention due to its low initial investment and ease of fabrication, high-vacuum thermal evaporation represents a promising alternative, with demonstrated efficiencies exceeding 20% for coevaporation^5–8^ and 26% in the case of sequential vacuum deposition.^9^ In particular, this deposition technique allows for improved conformality and homogeneity in the case of deposition over large-area^10,11^ and/or textured substrates,^12,13^ which is of particular relevance for tandem solar cells.

One of the challenges of perovskite coevaporation is the control over the deposition rates of the perovskite precursors, in particular those of organic salts such as methyl­ammonium iodide (MAI). For MAI, different studies suggest that its sublimation properties depend strongly on the impurity level,^14–16^ which can impact the perovskite formation. In addition, the poor adhesion of organics to the quartz crystal microbalances (QCMs) used to monitor the evaporation rates complicates the process and hinders the reproducibility. This shortcoming might be alleviated by a rigorous calibration and by monitoring the MAI rates indirectly through a QCM located at the substrate level.^16,17^ Other materials such as methyl­ammonium chloride (MACl) used in solution processing of perovskites^18–21^ and in the work on evaporated perovskite solar cells^22^ are found to be especially difficult to evaporate in a reproducible manner.^23^

In the literature, chloride (Cl) is often reported to enhance charge carrier transport properties in perovskite films, consequence of a reduction in the defect density.^2,18,20,21,24^ Another study on solution-processed perovskite reports a change in the grain growth dynamics when Cl is added to the precursor solution, resulting in apparent larger grains.^19^ However, the authors found that chloride assists only the perovskite formation, as it does not persist in the crystal structure after annealing. Instead, it was found that Cl leaves the film during the annealing step in the form of MACl, possibly due to the low solubility of chloride in pure-iodide systems.^25^ Nevertheless, in the context of vacuum-deposited perovskites, the use of chloride has not been extensively investigated. This is partly due to the fact that most organic ammonium chloride salts exhibit nonconstant sublimation behavior and are thus difficult to control in perovskite evaporation processes.

In this work, we present a simple approach for the controlled sublimation of methyl­ammonium-based organic compounds by pressing the precursor powder into a pellet. We show that the pellet can be sublimed the same way as the loose powder but with a reduced material consumption and improved control, as evidenced by a lower chamber pressure. Furthermore, pressing pellets of a mixture of different powders such as MAI and MACl makes it possible to evaporate multiple materials at the same time and incorporate a small amount of chloride into the perovskite film. This new approach is successfully demonstrated to deposit thin films of the wide-bandgap perovskite MAPb­(I_1–*x*_Br_*x*_)_3_ (hereafter referred to as MAPIBr for simplicity) with different concentrations of chloride, as determined by the MACl proportion in the pellet. We show that chloride is effectively incorporated in the perovskite lattice, where it enhances charge-transport properties and also increases the material’s bandgap. The modified vacuum-deposited MAPIBr:Cl perovskite is used as the active layer in a *p-i-n* solar cell, achieving a PCE of up to 17.9% for a 1.74 eV bandgap, outperforming the chloride-free counterpart with respect to their radiative efficiency limit. Finally, by reducing the bromide content to target a bandgap optimal for perovskite/silicon tandem applications (i.e., in the range of 1.65–1.7 eV),^26–28^ a PCE of 19.5% was achieved for a 1.66 eV bandgap when combining chloride inclusion with perovskite surface passivation.

We begin by comparing the deposition of MAPIBr perovskite films using MAI powder or a MAI pellet. The pellet is prepared by grinding the MAI powder using a mortar and pestle and pressing the resulting fine powder in a 10 mm-diameter die with a hydraulic press (more details can be found in the Experimental method in the Supporting Information). The MAI (in either powder or pellet form) is then placed inside an alumina crucible and heated in a high-vacuum chamber for sublimation. Simultaneously, the lead iodide (PbI_2_) and lead bromide (PbBr_2_) are cosublimed from a single source as a Pb­(I_1–*x*_Br_*x*_)_2_ mixture, as described previously.^29^

Figure 1a presents the MAI source temperature and corresponding chamber pressure as functions of the perovskite deposition time. Using the loose MAI powder, the pressure inside the chamber increases by one order of magnitude above that of the pellet during the initial heating stage. In addition, a lower temperature is required to reach the desired deposition rate when a powder is used. This is likely a consequence of the higher surface area of the powder, allowing it to sublime more easily. Additionally, MAI consumption is reduced by about 20 wt% when sublimed from the pellet, whereas it remains similar for the Pb­(I_1–*x*_Br_*x*_)_2_ source, as shown in Figure 1b.

**Figure 1:**
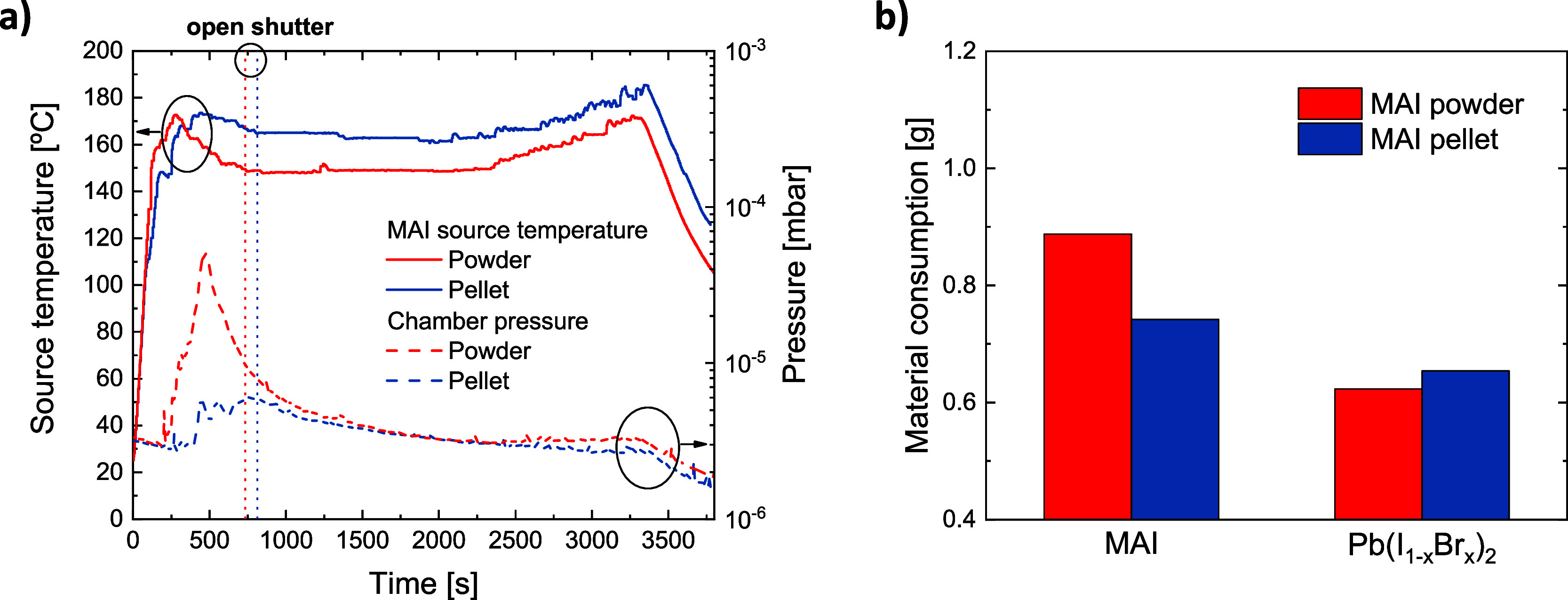
a) MAI source temperature and chamber pressure over MAPIBr deposition time using a MAI pellet and MAI powder for a targeted deposition rate of 1.8 Å/s at the substrate level. b) Material consumption of MAI and Pb­(I_1–*x*_Br_*x*_)_2_ precursors during those two depositions. Resulting films were both 500 nm thick.

As mentioned above, the inclusion of chloride has been reported to be beneficial for the properties of multi-halide perovskites. To explore its effect in a coevaporation process, we incorporated MACl in controlled amounts in the MAI pellet and sublimed it from a single source. We prepared 4 different pellets with different MACl content (by mass, wt%), namely 0, 5, 15, and 20 wt%. Those pellets were used as source material to sublime MAI:MACl alongside the Pb­(I_1–*x*_Br_*x*_)_2_ precursor with a 1:7 PbBr_2_:PbI_2_ ratio, to form perovskites with different amounts of chloride, referred to as MAPIBr, MAPIBrCl_5_, MAPIBrCl_15_, and MAPIBrCl_20_, respectively. Note that this nomenclature does not reflect the actual composition of the perovskite but rather refers to the precursors used to prepare it. We initially characterize 500-nm-thick perovskite films deposited on the hole transport material (HTL) used in our devices, as detailed later in the text. As shown in Figure 2a, the photoluminescence (PL) peak blue-shifts when the MACl content in the pellet is increased, from 752 nm for MAPIBr to 717 nm for MAPIBrCl_20_. This change indicates a bandgap increase of the corresponding perovskite from 1.65 to 1.73 eV and also suggests that chloride is incorporated into the crystal lattice, as smaller halides typically increase the bandgap.^30^ The X-ray diffraction (XRD) patterns depicted in Figure 2b are compatible with a tetragonal perovskite phase and a coexisting PbI_2_ phase, as evidenced by the peak at 2θ = 12.6°. Interestingly, the perovskite peaks shift to wider angles as the MACl content increases (Figure 2c), indicating a reduction of the lattice constant. This suggests that Cl, with an atomic radius (181 pm) smaller than that of iodide (220 pm), is incorporated within the perovskite structure. The presence of bromide might also help bridge the radii difference between iodide and chloride and promote the chloride solubility inside the crystal structure. The addition of chloride also seems to improve the crystallinity of the film (higher signal-to-noise ratio) as well as the conversion of PbI_2_, although the trend is not monotonic. In fact, the changes observed in the XRD patterns could also be influenced by a difference in the film orientation, as the intensity of the peaks (I) around 2θ ≈ 20° and 25° decreases with increasing MACl content, and their ratios to the most intense reflection at 2θ ≈ 14.2° change substantially. Using pseudocubic indexing, the peak at 2θ ≈ 14.2° corresponds to the (100) planes and those at ∼20.0° and ∼25.0° to the (110) and (111) planes, respectively. The progressive decrease of I(110) and I­(111) relative to I(100) with increasing MACl indicates a stronger (100) out-of-plane texture in the films. The presence of Cl in the perovskite film was confirmed by X-ray fluorescence (XRF) (Table S1). However, a precise analysis of the Cl concentration is difficult to perform quantitatively, as the main chloride peak (K_α1_ = 2.623 eV) overlaps with a secondary peak of lead (M_γ1_ = 2.658 eV). The fact that chloride seems to be incorporated within the perovskite crystal structure is in contrast with other reports, where chloride assists the perovskite crystallization by affecting the grain size but does not remain in the crystal structure.^19,31^

**Figure 2:**
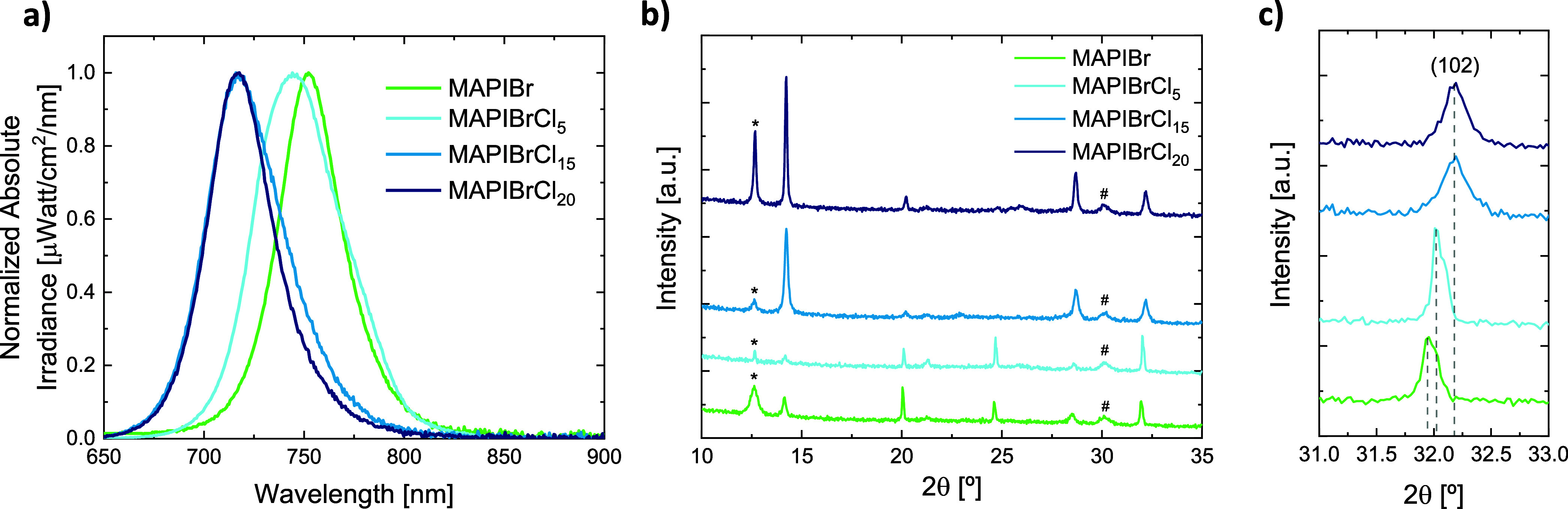
a) Normalized PL spectra of MAPIBr films containing different amounts of Cl. b) XRD patterns of the same perovskite films, where reflections corresponding to PbI_2_ and ITO are highlighted with * and #, respectively. c) Zoom of the XRD patterns around the (102) peak to showcase the peak shift.

In our case, scanning electron microscopy (SEM) images in Figure 3a–d suggest that the average domain size remains unchanged upon the addition of chloride, although the MAPIBrCl_20_ sample shows grains with sharper features. The films are composed of relatively small grains, which is typical for coevaporated perovskites, but the cross-sectional images confirm that the layers are continuous and flat. Moreover, small features visible in the MAPIBr film disappear in the presence of MACl, indicating that chloride does impact the growth and crystallization of the film.

**Figure 3:**
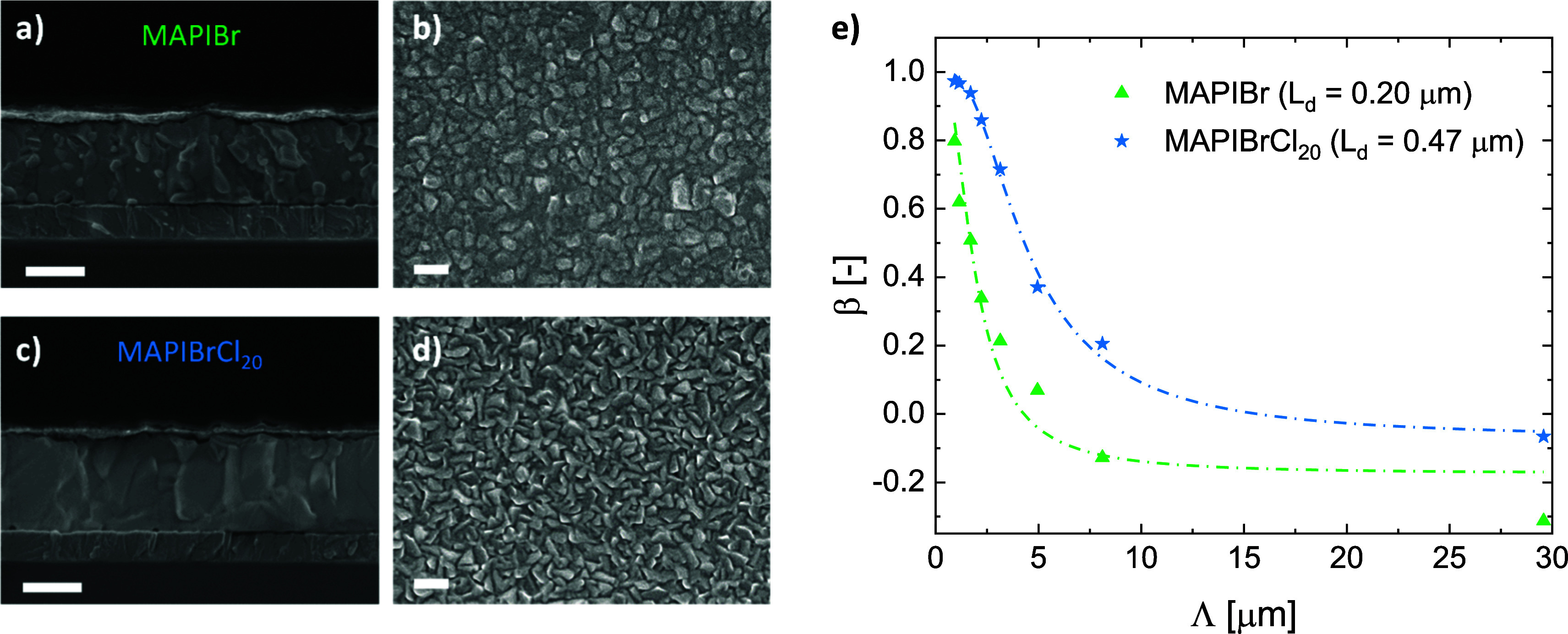
SEM images of (a-b) MAPIBr and (c-d) MAPIBrCl_20_ samples; scale bar is 300 nm in all micrographs. The cross-sectional images, taken on full devices, reveal the presence of small features in the MAPIBr perovskite film that disappear in the presence of MACl. e) SSPG measurement of MAPIBr films with and without chloride in the organic pellet. Fitted curves are indicated by the dash-dotted lines. Results indicate that the lateral diffusion length of the film doped with chloride is more than twice that of the reference sample.

It is worth noting that increasing the MACl content to above 15% does not lead to a further blue-shift of the PL peak nor a change in the XRD peak position, which might indicate that the chloride intake is a self-limiting process. A possible explanation is that Cl incorporation into the MAPbI_3_ lattice is limited by thermodynamic solubility: the ionic size mismatch between I^–^ and Cl^–^ makes higher substitution energetically unfavorable, so that additional Cl is more likely to segregate or form secondary phases rather than enter the perovskite lattice. Similar solubility limits for Cl incorporation have been suggested in earlier experimental and theoretical studies.^20,32,33^

To evaluate the charge transport properties of the films, we employed the steady-state photocarrier grating (SSPG)^34,35^ and the moving grating (MGT)^36,37^ techniques. Both techniques were developed to study amorphous silicon, but they have been also successfully applied to perovskite semiconductors.^38–41^ A brief explanation of the working principle of each technique is given in the Supporting Information. The main parameter extracted from SSPG is the diffusion length (L_d_) of the minority carriers, using the expression by Ritter et al.^34,35^ to fit the β parameter (which is the ratio of photocurrents with and without grating illumination) as a function of the grating period Λ. Considering the absorption coefficient at the wavelength of the laser (here 633 nm) and the sample thickness, we adjusted the laser intensity to achieve a generation rate of approximately 10^21^ cm^–3^ s^–1^, equivalent to 1-sun conditions. We analyzed the two extremes of our sample series, MAPIBr and MAPIBrCl_20_, and good fits were obtained for both samples, as shown in Figure 3e. Adding a minor amount of Cl to the perovskite results in a significant increase in the diffusion length, from 200 to 470 nm, implying better bulk transport properties. While these diffusion lengths are lower than values typically reported for perovskites, we note that the SSPG technique gives the lateral (in-plane) diffusion length, whereas in the case of solar cells, the diffusion length of interest is in the perpendicular direction (out-of-plane). This general discrepancy between low lateral diffusion lengths and efficient carrier collection in PSCs was recently rationalized in a study from Cho et al.^42^

Additionally, as shown in Figure S2, MGT measurements show that chloride incorporation not only enhances the transport properties but also changes the semiconductor character from p- to n-type. Although the microscopic origin for this change is not yet fully understood, one possible explanation is a modification of the defect distribution, shifting the Fermi level closer to the conduction band. In a *p-i-n* configuration, this change is relevant because in an n-type absorber, the minority carriers (holes) are generated closer to the hole transport layer at the front interface, which can facilitate their extraction.

The MAPIBr films with and without Cl were used to fabricate fully evaporated *p-i-n* PSCs with the following stack, represented in Figure 4a: antireflective coating (ARC)/​Glass/​ITO/​CS90112/​TaTm/​perovskite/​C_60_/​BCP/Ag, where CS90112 is 2,2′,2″-(cyclo­propane-1,2,3-triylidene)­tris­(2-(cyano­tetra­fluoro­phenyl)­aceto­nitrile), TaTm is *N*4,*N*4,*N*4″,*N*4″-tetra­([1,1′-biphenyl]-4-yl)-[1,1′:4′,1″-terphenyl]-4,4″-diamine), and BCP is batho­cuproine (further details about device fabrication can be found in the Experimental method in the Supporting Information). The perovskites, namely, MAPIBr and MAPIBrCl_20_, were employed in this device stack to evaluate the effect of chloride on the device performance. EQE spectra and *J–V* curves obtained under simulated AM1.5G light spectrum are depicted in Figure 4b and c, respectively, with the corresponding statistics in Figure 4d. As expected from the blue-shifted PL signal shown in Figure 2a, the effective bandgap derived from the EQE increases from 1.65 to 1.74 eV (see Figure S3 for derivation), in good agreement with the PL peak position. Consequently, the *V*_oc_ increases from 1.13 to 1.17 V with higher chloride content and for the same reason, the *J*_sc_ decreases from 19.8 to 18.3 mA/cm^2^. Interesting is the increase in fill factor (FF) from 78.8 to 81.0% with increasing Cl composition, which is attributed to the higher diffusion length observed from the SSPG analysis (Figure 3e). The n-type character revealed by MGT (Figure S2) may also contribute to the increase in the fill factor. In *p-i-n* solar cells, if the absorber is n-type, the photogenerated holes are efficiently extracted at the front interface while electrons, as majority carriers, are collected at the rear, leading to improved charge collection and higher FF. While the PCE value of the MAPIBrCl_20_ devices is on average slightly lower than the chloride-free MAPIBr reference (i.e., 17.4 instead of 17.7%), the former perovskite presents better performances when compared to its radiative limit (i.e., the maximum theoretical *J–V* values for a given bandgap^43^). As shown in Figure S4, this improvement is due to a better *J*_sc_ and FF with respect to the detailed balance values for a 1.74 eV absorber, reaching 60% of the maximum potential PCE and exceeding that of the chloride-free PSCs. On the downside, the relative percentage of the *V*_oc_ limit becomes slightly lower, possibly as a consequence of the larger bandgap which tends to show greater *V*_oc_ losses.^44^

**Figure 4:**
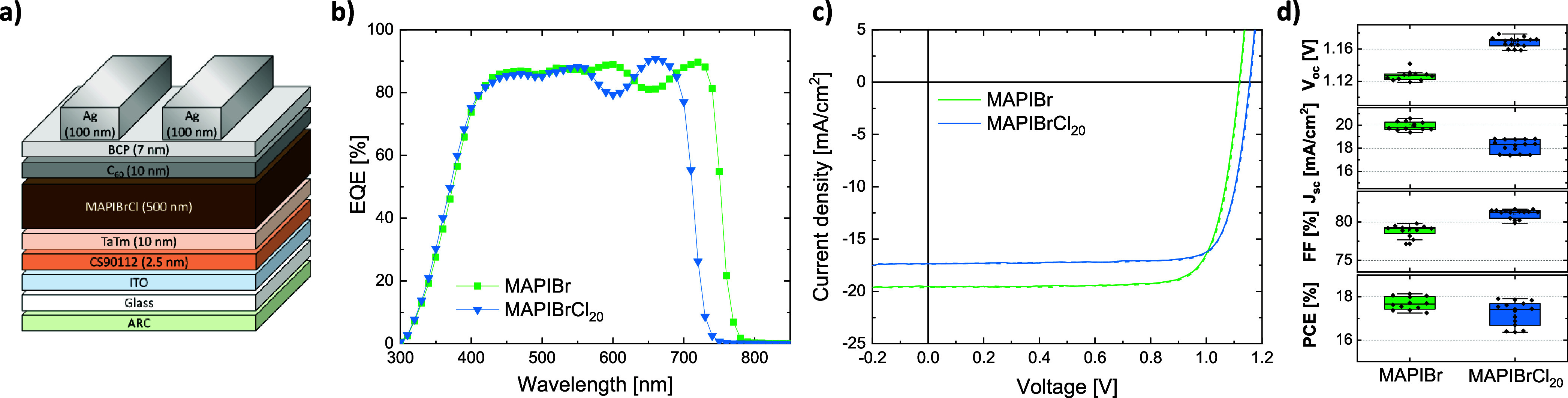
a) Device stack employed in this work. b) EQE of MAPIBr PSCs without and with MACl in the MAI organic pellet. c) Representative *J–V* curves (forward and reverse scan in solid and dashed line, respectively) of those same devices. d) Corresponding statistics of PV parameters from reverse scan.

The incorporation of chloride in the perovskite resulted in a bandgap which is too large for application in efficient perovskite-silicon tandem devices, where an optimal value of approximately 1.65–1.68 eV is required.^26–28^ Hence, we reduced the bromide content from a PbBr_2_:PbI_2_ ratio of 1:7 (i.e., 12.5 wt%) to 1:9 (i.e., 10 wt%), while keeping the MACl at 20 wt% in the pellet. This resulted in a new bandgap of 1.66 eV (derived from EQE in Figure S5b), within the targeted range, and averaged PCE of 18.3% (1.14 V *V*_oc_, 20.18 mA/cm^2^ *J*_sc_, and 79.5% FF), as shown in Figure 5a and b. Furthermore, an additional passivation step was introduced by thermally evaporating 1 nm of ethylene­diammonium diiodide (EDAI_2_) on top of the perovskite surface. This thin layer reduced drastically the nonradiative recombination induced by the deposition of the subsequent C_60_ layer, as seen from the PL intensity shown in Figure S5c, and led to a *V*_oc_ gain of 40 mV and a 1.5% (absolute) increase in FF. This surface passivation effect is in line with other reports in the literature where the same or similar molecules were used for the same purpose.^45–49^ Overall, devices of 19.3% PCE on average could be achieved (1.18 V *V*_oc_, 20.20 mA/cm^2^ *J*_sc_, and 81.0% FF) with a champion device of 19.5%. Preliminary thermal stability tests (85 °C in the dark) are shown in Figure S6. The *V*_oc_ remained constant and the *J*_sc_ decreased by less than 5%, while the main performance loss was due to a reduction in fill factor (Figure S6c), suggesting that degradation is more related to transport layers or interfaces than to the perovskite film itself.

**Figure 5:**
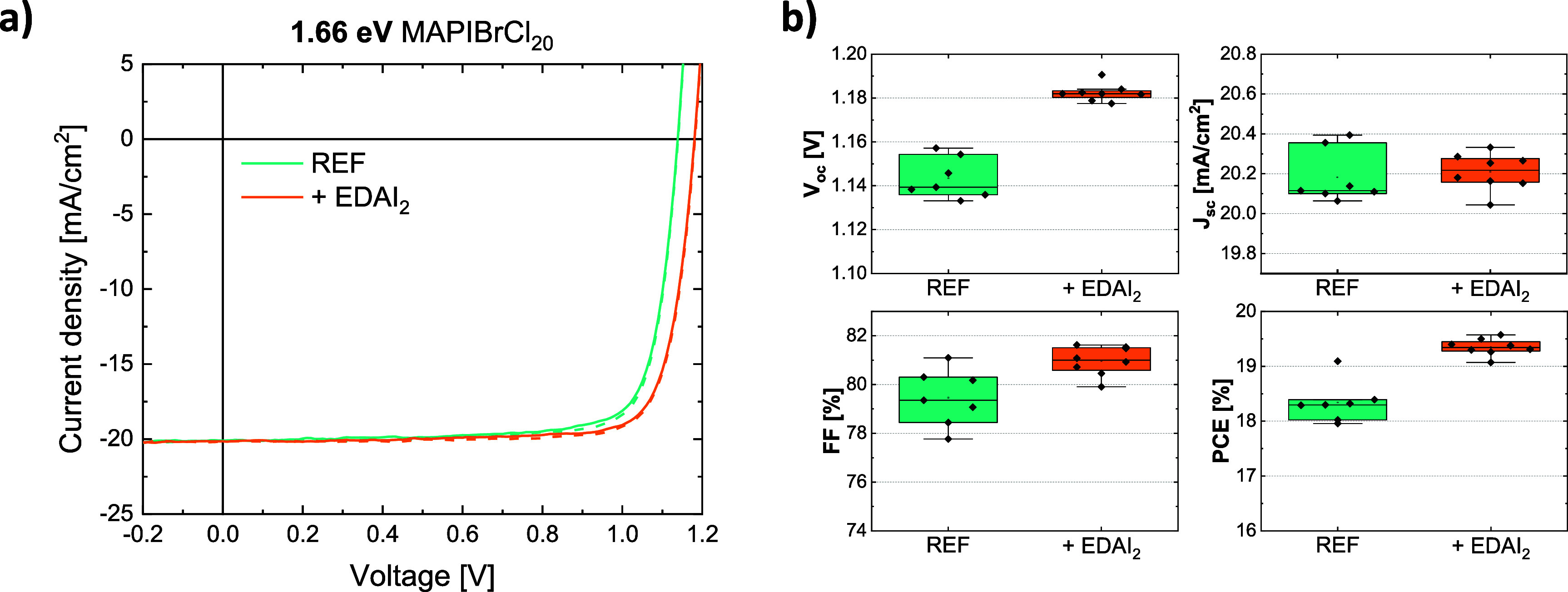
a) Representative *J–V* curves (forward and reverse scan in solid and dashed lines, respectively) of MAPIBrCl_20_ devices containing a lower amount of bromide, with and without EDAI_2_ passivation. b) Device statistics (from reverse scan).

In conclusion, we propose a novel approach to sublime organic materials by pressing them into a pellet, leading to better control over the evaporation process and reduced material consumption. In addition, other organic compounds can also be introduced in the pellet in varying amounts, adding novel components to the perovskite without complicating the deposition process. Notably, MACl was used in conjunction with MAI to obtain the triple-halide MAPIBrCl perovskite with enhanced charge-carrier transport properties, leading to an FF improvement in PSC devices and to 60% of the theoretical maximum efficiency for that bandgap. Lastly, by reducing the bromide content and passivating the perovskite surface with a thin EDAI_2_ layer, fully vacuum-processed devices with a bandgap of 1.66 eV and PCE of up to 19.5% were obtained.

## Supplementary Material



## Notes

Experimental method, XRF data, MGT analysis of MAPIBr and MAPIBrCl_20_ films, effective bandgap derivation from EQE measurements, device performance with respect to the respective radiative limit, and PL signal with and without EDAI_2_ passivation. (PDF)

## References

[1] De
Wolf S., Holovsky J., Moon S.-J., Löper P., Niesen B., Ledinsky M., Haug F.-J., Yum J.-H., Ballif C. (2014). Organometallic Halide Perovskites: Sharp Optical Absorption
Edge and Its Relation to Photovoltaic Performance. J. Phys. Chem. Lett..

[2] Stranks S. D., Eperon G. E., Grancini G., Menelaou C., Alcocer M. J. P., Leijtens T., Herz L. M., Petrozza A., Snaith H. J. (2013). Electron-Hole
Diffusion Lengths Exceeding 1 Micrometer in an Organometal Trihalide
Perovskite Absorber. Science.

[3] Eperon G. E., Stranks S. D., Menelaou C., Johnston M. B., Herz L. M., Snaith H. J. (2014). Formamidinium Lead
Trihalide: A Broadly Tunable Perovskite
for Efficient Planar Heterojunction Solar Cells. Energy Environ. Sci..

[4] Best Research-Cell Efficiency Chart. https://www.nrel.gov/pv/cell-efficiency.html (accessed 2024-07-22).

[5] Momblona C., Gil-Escrig L., Bandiello E., Hutter E. M., Sessolo M., Lederer K., Blochwitz-Nimoth J., Bolink H. J. (2016). Efficient Vacuum
Deposited P-i-n and n-i-p Perovskite Solar Cells Employing Doped Charge
Transport Layers. Energy Environ. Sci..

[6] Roß M., Gil-Escrig L., Al-Ashouri A., Tockhorn P., Jošt M., Rech B., Albrecht S. (2020). Co-Evaporated p-i-n Perovskite Solar
Cells beyond 20% Efficiency: Impact of Substrate Temperature and Hole-Transport
Layer. ACS Appl. Mater. Interfaces.

[7] Feng J., Jiao Y., Wang H., Zhu X., Sun Y., Du M., Cao Y., Yang D., Liu S. (2021). High-Throughput Large-Area
Vacuum Deposition for High-Performance Formamidine-Based Perovskite
Solar Cells. Energy Environ. Sci..

[8] Rodkey N., Zanoni K. P. S., Piot M., Dreessen C., Grote R., Carroy P., Sebastian
Alonso J. E., Paliwal A., Muñoz D., Bolink H. J. (2024). Efficient Micrometer Thick Bifacial Perovskite Solar
Cells. Adv. Energy Mater..

[9] Zhou J., Tan L., Liu Y., Li H., Liu X., Li M., Wang S., Zhang Y., Jiang C., Hua R., Tress W., Meloni S., Yi C. (2024). Highly Efficient and
Stable Perovskite Solar Cells via a Multifunctional Hole Transporting
Material. Joule.

[10] Li J., Wang H., Chin X. Y., Dewi H. A., Vergeer K., Goh T. W., Lim J. W. M., Lew J. H., Loh K. P., Soci C., Sum T. C., Bolink H. J., Mathews N., Mhaisalkar S., Bruno A. (2020). Highly Efficient Thermally Co-Evaporated
Perovskite Solar Cells and Mini-Modules. Joule.

[11] Li J., Dewi H. A., Wang H., Lew J. H., Mathews N., Mhaisalkar S., Bruno A. (2020). Design of Perovskite Thermally Co-Evaporated
Highly Efficient Mini-Modules with High Geometrical Fill Factors. Sol. RRL.

[12] Roß M., Severin S., Stutz M. B., Wagner P., Köbler H., Favin-Lévêque M., Al-Ashouri A., Korb P., Tockhorn P., Abate A., Stannowski B., Rech B., Albrecht S. (2021). Co-Evaporated Formamidinium Lead
Iodide Based Perovskites with 1000 h Constant Stability for Fully
Textured Monolithic Perovskite/Silicon Tandem Solar Cells. Adv. Energy Mater..

[13] Gil-Escrig L., Roß M., Sutter J., Al-Ashouri A., Becker C., Albrecht S. (2021). Fully Vacuum-Processed Perovskite
Solar Cells on Pyramidal Microtextures. Sol.
RRL.

[14] Borchert J., Levchuk I., Snoek L. C., Rothmann M. U., Haver R., Snaith H. J., Brabec C. J., Herz L. M., Johnston M. B. (2019). Impurity
Tracking Enables Enhanced Control and Reproducibility of Hybrid Perovskite
Vapor Deposition. ACS Appl. Mater. Interfaces.

[15] Roß M., Stutz M. B., Albrecht S. (2022). Revealing
the Role of Methylammonium
Iodide Purity on the Vapor-Phase Deposition Process of Perovskites. Sol. RRL.

[16] Zanoni K. P. S., Martínez-Goyeneche L., Dreessen C., Sessolo M., Bolink H. J. (2023). Photovoltaic Devices Using Sublimed Methylammonium
Lead Iodide Perovskites: Long-Term Reproducible Processing. Sol. RRL.

[17] Kim B.-S., Gil-Escrig L., Sessolo M., Bolink H. J. (2020). Deposition Kinetics
and Compositional Control of Vacuum-Processed CH3NH3PbI3 Perovskite. J. Phys. Chem. Lett..

[18] Xu J., Boyd C. C., Yu Z. J., Palmstrom A. F., Witter D. J., Larson B. W., France R. M., Werner J., Harvey S. P., Wolf E. J., Weigand W., Manzoor S., van Hest M. F. A. M., Berry J. J., Luther J. M., Holman Z. C., McGehee M. D. (2020). Triple-Halide Wide–Band Gap Perovskites with
Suppressed Phase Segregation for Efficient Tandems. Science.

[19] Chen B., Yu Z., Liu K., Zheng X., Liu Y., Shi J., Spronk D., Rudd P. N., Holman Z., Huang J. (2019). Grain Engineering
for Perovskite/Silicon Monolithic Tandem Solar Cells with Efficiency
of 25.4%. Joule.

[20] Colella S., Mosconi E., Fedeli P., Listorti A., Gazza F., Orlandi F., Ferro P., Besagni T., Rizzo A., Calestani G., Gigli G., De Angelis F., Mosca R. (2013). MAPbI3-xClx Mixed Halide
Perovskite for Hybrid Solar Cells: The Role
of Chloride as Dopant on the Transport and Structural Properties. Chem. Mater..

[21] Xie F., Chen C.-C., Wu Y., Li X., Cai M., Liu X., Yang X., Han L. (2017). Vertical Recrystallization for Highly
Efficient and Stable Formamidinium-Based Inverted-Structure Perovskite
Solar Cells. Energy Environ. Sci..

[22] Dreessen C., Zanoni K. P. S., Gil-Escrig L., Rodkey N., Khan J. I., Laquai F., Sessolo M., Roldán-Carmona C., Bolink H. J. (2024). When JV Curves Conceal
Material Improvements: The Relevance
of Photoluminescence Measurements in the Optimization of Perovskite
Solar Cells. Adv. Opt. Mater..

[23] Leyden M. R., Škorjanc V., Miaskiewicz A., Severin S., Maniyarasu S., Gries T., Beckedahl J., Scheler F., Simmonds M., Holzhey P., Kurpiers J., Korte L., Roß M., Albrecht S. (2024). Loading Precursors into Self-Assembling Contacts for
Improved Performance and Process Control in Evaporated Perovskite
Solar Cells. Sol. RRL.

[24] Wehrenfennig C., Eperon G. E., Johnston M. B., Snaith H. J., Herz L. M. (2014). High Charge
Carrier Mobilities and Lifetimes in Organolead Trihalide Perovskites. Adv. Mater..

[25] Unger E. L., Bowring A. R., Tassone C. J., Pool V. L., Gold-Parker A., Cheacharoen R., Stone K. H., Hoke E. T., Toney M. F., McGehee M. D. (2014). Chloride in Lead Chloride-Derived Organo-Metal Halides
for Perovskite-Absorber Solar Cells. Chem. Mater..

[26] Leijtens T., Bush K. A., Prasanna R., McGehee M. D. (2018). Opportunities and
Challenges for Tandem Solar Cells Using Metal Halide Perovskite Semiconductors. Nat. Energy.

[27] Jošt M., Köhnen E., Morales-Vilches A. B., Lipovšek B., Jäger K., Macco B., Al-Ashouri A., Krč J., Korte L., Rech B., Schlatmann R., Topič M., Stannowski B., Albrecht S. (2018). Textured Interfaces
in Monolithic Perovskite/Silicon Tandem Solar Cells: Advanced Light
Management for Improved Efficiency and Energy Yield. Energy Environ. Sci..

[28] Aydin E., Allen T. G., De Bastiani M., Xu L., Ávila J., Salvador M., Van Kerschaver E., De Wolf S. (2020). Interplay between Temperature
and Bandgap Energies on the Outdoor Performance of Perovskite/Silicon
Tandem Solar Cells. Nat. Energy.

[29] Gil-Escrig L., Susic I., Doğan İ., Zardetto V., Najafi M., Zhang D., Veenstra S., Sedani S., Arikan B., Yerci S., Bolink H. J., Sessolo M. (2023). Efficient and Thermally
Stable Wide Bandgap Perovskite Solar Cells by Dual-Source Vacuum Deposition. Adv. Funct. Mater..

[30] Protesescu L., Yakunin S., Bodnarchuk M. I., Krieg F., Caputo R., Hendon C. H., Yang R. X., Walsh A., Kovalenko M. V. (2015). Nanocrystals
of Cesium Lead Halide Perovskites (CsPbX3, X = Cl, Br, and I): Novel
Optoelectronic Materials Showing Bright Emission with Wide Color Gamut. Nano Lett..

[31] Dong Q., Yuan Y., Shao Y., Fang Y., Wang Q., Huang J. (2015). Abnormal Crystal Growth in CH3NH3PbI3–xClx Using a Multi-Cycle
Solution Coating Process. Energy Environ. Sci..

[32] Osherov A., Feldman Y., Kaplan-Ashiri I., Cahen D., Hodes G. (2020). Halide Diffusion
in MAPbX3: Limits to Topotaxy for Halide Exchange in Perovskites. Chem. Mater..

[33] Howlader A. H., Uddin A. (2023). Progress and Challenges of Chloride–Iodide
Perovskite Solar
Cells: A Critical Review. Nanomanufacturing.

[34] Ritter D., Zeldov E., Weiser K. (1986). Steady-state
Photocarrier Grating
Technique for Diffusion Length Measurement in Photoconductive Insulators. Appl. Phys. Lett..

[35] Ritter D., Zeldov E., Weiser K. (1988). Ambipolar Transport in Amorphous
Semiconductors in the Lifetime and Relaxation-Time Regimes Investigated
by the Steady-State Photocarrier Grating Technique. Phys. Rev. B.

[36] Haken U., Hundhausen M., Ley L. (1993). Moving Grating Technique:
A New Method
for the Determination of Electron and Hole Mobilities and Their Lifetime. Appl. Phys. Lett..

[37] Haken U., Hundhausen M., Ley L. (1995). Analysis of the Moving-Photocarrier-Grating
Technique for the Determination of Mobility and Lifetime of Photocarriers
in Semiconductors. Phys. Rev. B.

[38] Ventosinos F., Koffman-Frischknecht A., Herrera W., Senno M., Caram J., Perez M. D., Schmidt J. A. (2020). Estimation of Carrier Mobilities
and Recombination Lifetime in Halide Perovskites Films Using the Moving
Grating Technique. J. Phys. Appl. Phys..

[39] Ventosinos F., Moeini A., Pérez-Del-Rey D., Bolink H. J., Schmidt J. A. (2022). Density of States within the Bandgap
of Perovskite
Thin Films Studied Using the Moving Grating Technique. J. Chem. Phys..

[40] Levine I., Gupta S., Brenner T. M., Azulay D., Millo O., Hodes G., Cahen D., Balberg I. (2016). Mobility–Lifetime
Products in MAPbI3 Films. J. Phys. Chem. Lett..

[41] Longeaud C. (2020). Study of Transport
Parameters and Defect States in Thin Film Perovskites under Different
Environments – Air or Vacuum – and after Light-Soaking. EPJ. Photovolt..

[42] Cho C., Feldmann S., Yeom K. M., Jang Y.-W., Kahmann S., Huang J.-Y., Yang T. C., Khayyat M. N. T., Wu Y.-R., Choi M., Noh J. H., Stranks S. D., Greenham N. C. (2022). Efficient
Vertical Charge Transport in Polycrystalline Halide Perovskites Revealed
by Four-Dimensional Tracking of Charge Carriers. Nat. Mater..

[43] Rühle S. (2016). Tabulated
Values of the Shockley–Queisser Limit for Single Junction Solar
Cells. Sol. Energy.

[44] Caprioglio P., Smith J. A., Oliver R. D. J., Dasgupta A., Choudhary S., Farrar M. D., Ramadan A. J., Lin Y.-H., Christoforo M. G., Ball J. M., Diekmann J., Thiesbrummel J., Zaininger K.-A., Shen X., Johnston M. B., Neher D., Stolterfoht M., Snaith H. J. (2023). Open-Circuit and
Short-Circuit Loss
Management in Wide-Gap Perovskite p-i-n Solar Cells. Nat. Commun..

[45] Chen H., Maxwell A., Li C., Teale S., Chen B., Zhu T., Ugur E., Harrison G., Grater L., Wang J., Wang Z., Zeng L., Park S. M., Chen L., Serles P., Awni R. A., Subedi B., Zheng X., Xiao C., Podraza N. J., Filleter T., Liu C., Yang Y., Luther J. M., De Wolf S., Kanatzidis M. G., Yan Y., Sargent E. H. (2023). Regulating Surface Potential Maximizes Voltage in All-Perovskite
Tandems. Nature.

[46] Liu C., Yang Y., Chen H., Xu J., Liu A., Bati A. S. R., Zhu H., Grater L., Hadke S. S., Huang C., Sangwan V. K., Cai T., Shin D., Chen L. X., Hersam M. C., Mirkin C. A., Chen B., Kanatzidis M. G., Sargent E. H. (2023). Bimolecularly Passivated
Interface
Enables Efficient and Stable Inverted Perovskite Solar Cells. Science.

[47] Wang J., Zeng L., Zhang D., Maxwell A., Chen H., Datta K., Caiazzo A., Remmerswaal W. H. M., Schipper N. R. M., Chen Z., Ho K., Dasgupta A., Kusch G., Ollearo R., Bellini L., Hu S., Wang Z., Li C., Teale S., Grater L., Chen B., Wienk M. M., Oliver R. A., Snaith H. J., Janssen R. A. J., Sargent E. H. (2024). Halide Homogenization for Low Energy
Loss in 2-eV-Bandgap Perovskites and Increased Efficiency in All-Perovskite
Triple-Junction Solar Cells. Nat. Energy.

[48] Hu S., Otsuka K., Murdey R., Nakamura T., Truong M. A., Yamada T., Handa T., Matsuda K., Nakano K., Sato A., Marumoto K., Tajima K., Kanemitsu Y., Wakamiya A. (2022). Optimized Carrier Extraction
at Interfaces for 23.6%
Efficient Tin–Lead Perovskite Solar Cells. Energy Environ. Sci..

[49] Hu S., Pascual J., Liu W., Funasaki T., Truong M. A., Hira S., Hashimoto R., Morishita T., Nakano K., Tajima K., Murdey R., Nakamura T., Wakamiya A. (2022). A Universal Surface Treatment for p–i–n
Perovskite Solar Cells. ACS Appl. Mater. Interfaces.

