# Supplementary material for: Vacuum Deposition of Triple-Halide Wide-Bandgap Perovskites Enabled by Sublimation of Mixed Organic-Halide Pellets
Source: ACS Mater Lett. 2025 Oct 16;7(11):3692–8. doi: 10.1021/acsmaterialslett.5c01161 (PMC12587446; doi:10.1021/acsmaterialslett.5c01161)
Supplement: Supplementary file 1 [file tz5c01161_si_001.pdf]

SUPPLEMENTARY INFORMATION

Vacuum Deposition of Triple-Halide Wide-Bandgap Perovskites Enabled by Sublimation of Mixed Organic-Halide Pellets

Manuel Piot[1], Lidón Gil-Escrig[1], Federico Ventosinos[1,2], Cristina Roldán-Carmona[1], Anna Robinson[3], Javier A. Schmidt[2], Michele Sessolo[1,*], Henk J. Bolink[1,*]

1. Instituto de Ciencia Molecular, Universidad de Valencia, Calle Catedrático José Beltrán 2, 46980 Paterna, Spain

2. Instituto de Física del Litoral (IFIS-Litoral), CONICET-UNL, Güemes 3450, S3000GLN, Santa Fe, Argentina

3. Oxford Photovoltaics Ltd, Unit 7/8 Mead Road, Yarnton, OX5 1QU, United Kingdom

Corresponding Authors:

Henk J. Bolink – henk.bolink@uv.es

Michele Sessolo – michele.sessolo@uv.es

Materials and methods

Materials

*N*4,*N*4,*N*4'',*N*4''-Tetra([1,1'-biphenyl]-4-yl)[1,1':4',1''-terphenyl]-4,4''-diamine (TaTm) and $PbBr_2$ were obtained from Tokyo Chemical Industry. Methylammonium iodide (MAI), 2,2',2''-(cyclopropane-1,2,3-triylidene)tris(2-(p-cyanotetrafluorophenyl)acetonitrile) (CS90112), bathocuproine (BCP) and PbI_2 were purchased from Luminescence Technology Corp. Fullerene (C_{60}) was obtained from M. Braun GmbH. Methylammonium chloride (MACl) and ethane-1,2-diammonium iodide (EDAI) were purchased from Greatcell.

Pellet fabrication

The desired proportion of MAI and MACl powders were ground inside of a nitrogen-filled glovebox using a mortar and pestle (for a total of about 1 gram of powder). The resulting fine powder mix was transferred into a pellet die (10 mm diameter) and compressed at 3 metric tons for 30 minutes using a hydraulic press. The resulting pellet was placed inside an alumina crucible and used as sublimation source.

Film and device preparation

ITO-coated glass substrates were subsequently cleaned with soap (2% Mucasol™ in water), water and isopropanol in an ultrasonic bath, followed by 15 min UV-ozone treatment. The substrates were transferred to a vacuum chamber integrated in a nitrogen-filled glovebox and evacuated to a pressure of 10^{-6} mbar for the charge extraction layers' deposition. In general, the deposition rate for the TaTm and C_{60} was 0.3 Å/s while the thinner CS90112 and BCP were deposited at 0.15 Å/s. Ag was evaporated in a separate vacuum chamber using aluminum boats as sources. The perovskite was evaporated in a dedicated vacuum chamber, equipped with four evaporation sources (M. Braun GmbH) with independent temperature controllers and shutters. All sources have a dedicated QCM sensor above and the materials are loaded in alumina crucibles. All sources were individually calibrated for their respective

materials and no cross-reading between materials is ensured by the relative position of the sources, shutters and sensors. The mixed halide $Pb(I_{1-x}Br_x)_2$ precursor was prepared by mixing in an alumina crucible the calculated amounts of PbI_2 and $PbBr_2$, and by heating them at 380 ºC for 5 minutes, when complete melting of the mixture is achieved. Typical sublimation temperatures for the precursors were approximately 150 ºC for MAI and 250 ºC for $Pb(I_{1-x}Br_x)_2$, which correspond to a deposition rate of 1.4 Å/s for the $Pb(I_{1-x}Br_x)_2$ source, controlled from dedicated sensors placed in proximity of the source, and of 1.8 Å/s at the substrate, obtained by controlling the temperature of the MAI source. All devices were coated with Al_2O_3 (30 nm) by atomic layer deposition (Arradiance's GEMStar XT Thermal ALD) prior to the characterization, which was carried out in ambient atmosphere.

Materials characterization

Optical absorption spectra were collected using fiber optics based Avantes Avaspec2048 Spectrometer. The photoluminescence spectra were measured with an Avantes Avaspec2048 spectrometer and films were illuminated with a diode laser of Integrated Optics, emitting at 515 nm. All spectra were collected with an integration time of 1 s. The XRD patterns were collected in Bragg-Brentano geometry on an Empyrean PANalytical powder diffractometer with a copper anode operated at 45 kV and 40 mA. Scanning Electron Microscopy (SEM) was performed with a high-resolution field-emission Hitachi SU8010 microscope operating at an accelerating voltage of 2 kV over platinum-metallized samples. Steady state photocarrier grating (SSPG) measurements were carried out using a He-Ne laser with 15 mW power and 632 nm wavelength. Samples for SSPG consists in perovskite films deposited on glass, coated with two 5 mm wide Au electrodes separated by a 0.5 mm gap. Using neutral density filters, a generation rate of $3 \cdot 10^{21}$ (cm^{-3} s^{-1}), which is close to 1 sun equivalent intensity, was obtained.

Moving Grating Technique (MGT)

For this measurement we use a 10 mW He-Ne laser with a wavelength of 633 nm. A scheme of the experimental setup can be found in the literature[1]. Acousto-optic modulators (AOMs)

were used to introduce a small frequency difference between the two optical paths. When two beams of equal polarization but different frequencies impinge on the sample, they create an interference pattern that moves at a constant speed proportional to the frequency difference and the grating period. This movement of the illumination pattern generates a short-circuit current density in a sample with coplanar contacts, even in the absence of an external electric field, as explained in the cited works.[1,2] By measuring the short-circuit current density as a function of the speed of the illumination pattern, a plot can be generated from which transport parameters of the sample, such as mobilities and the mutual recombination lifetime, can be estimated. Typical MGT curves (**Figure S1**) are characterized by a peak in the current density, that reaches a maximum at a characteristic speed related to the recombination lifetime of the carriers. If the grating speed is reversed, the behavior of the curve remains the same, but the sign of the current is inverted.

To determine the semiconductor type, a straightforward method is to compare the sample curve with that of a known material. In this case, hydrogenated amorphous silicon (a-Si:H), which is known to be an n-type semiconductor (due to the presence of oxygen and nitrogen contaminants which shift the Fermi level closer to the conduction band), was used as a reference. **Figure S1** shows a measurement of an a-Si:H sample, displaying a clear positive peak for negative velocities. This indicates that samples with a positive peak in the negative x-axis are n-type, while those with a positive peak in the positive x-axis are p-type. Therefore, as shown in **Figure S2**, the reference MAPIBr sample is p-type, while the $MAPIBrCl_{20}$ is n-type.

Steady State Photocarrier Grating technique (SSPG)

SSPG data was measured using a 20 mW He-Ne laser with a wavelength of 633 nm divided using a 90/10 beam splitter. The strong beam passes through a $\lambda/2$ plate that can change between vertical (yielding interference) or horizontal (no interference on the sample) polarizations. The weak beam is directed to the sample through a moveable mirror that later directs to 8 fixed mirrors. This configuration allows us to vary the grating period between 0.8 to 30 microns. A scheme of the experimental setup can be found in the literature.[3] In this technique the central idea is to calculate the parameter called β for several grating period

values (8 in our case). β is calculated as the ratio between the photocurrent measured when an interference pattern is present and when there is not (homogeneous illumination). As explained in the original publication[4], in the limit of very short grating periods, even if there are optical fringes, electrically, the diffusion of carriers homogenizes the distribution of carriers, making β closer to 1. In the high grating period limit, diffusion of carriers can no longer homogenize the distribution, making zones of higher and lower resistivity, which diminishes the current, decreasing β. To obtain an estimation of the diffusion length of minority carriers we make use of Ritter-Weiser-Zeldov formula[4] for fitting the data in the low voltage regime.

Device characterization

JV curves were recorded using a Keithley 2612A SourceMeter in a -0.2 and 1.2 V voltage range, with 0.01 V steps and integrating the signal for 20 ms after a 10 ms delay, corresponding to a scan speed of about 0.1 V s^{-1}. The devices were illuminated under a Wavelabs Sinus 70 LED solar simulator. The light intensity was adjusted before every measurement using a calibrated silicon reference diode. EQE measurements were performed on a QE-R system from Enlitech. The system was calibrated and the solar spectrum mismatch was corrected using a calibrated silicon reference cell. The EQE was measured as a function of wavelength from 300 nm to 850 nm in 10 nm steps.

Table S1. XRF data of MAPIBr and $MAPIBrCl_{20}$ films

	Density [g/m²]	Pb [%at]	I [%at]	Br [%at]	Cl [%at]	Si [%mass]	O [%mass]	C [%mass]
**MAPIBr #1**	2.683	3.614	9.853	1.049	0.011	43	56.5	0.5
**MAPIBr #2**	2.71	3.609	9.833	1.04	0.01	43	56.5	0.5
**$MAPIBrCl_{20}$ #1**	2.783	3.447	9.352	1.037	0.49	43	56.5	0.5
**$MAPIBrCl_{20}$ #2**	2.753	3.521	9.451	1.057	0.428	43	56.5	0.5

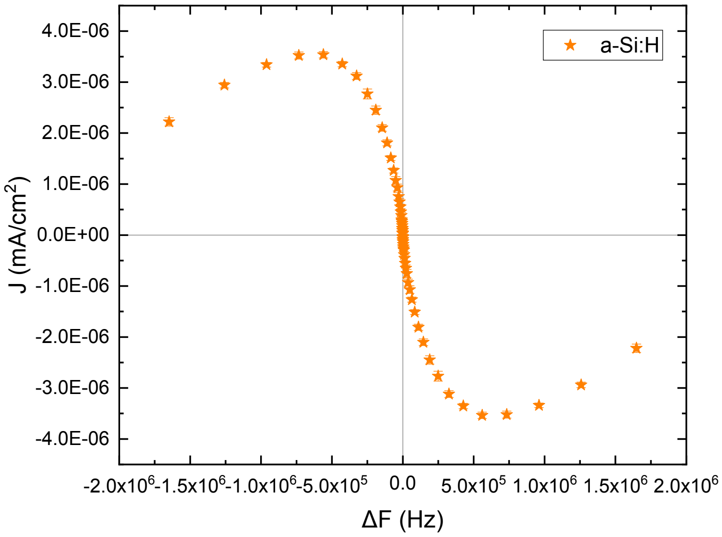

Figure S1. Moving Grating Technique measurement of an a-Si:H sample, known to be n-type. Thus, perovskite films with positive maximum peak in the negative axis are n-type semiconductors, while they are p-type if the positive peak is in the positive x axis.

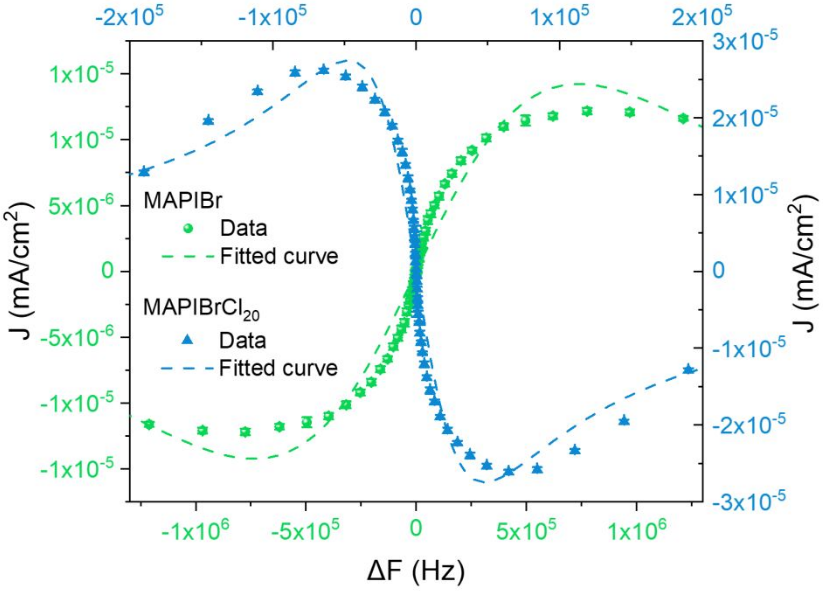

Figure S2. MGT of MAPIBr films without and with MACl in the MAI organic pellet. The change of sign indicates a difference in semiconductor type, going from p- to n-type when chloride is introduced in the film.

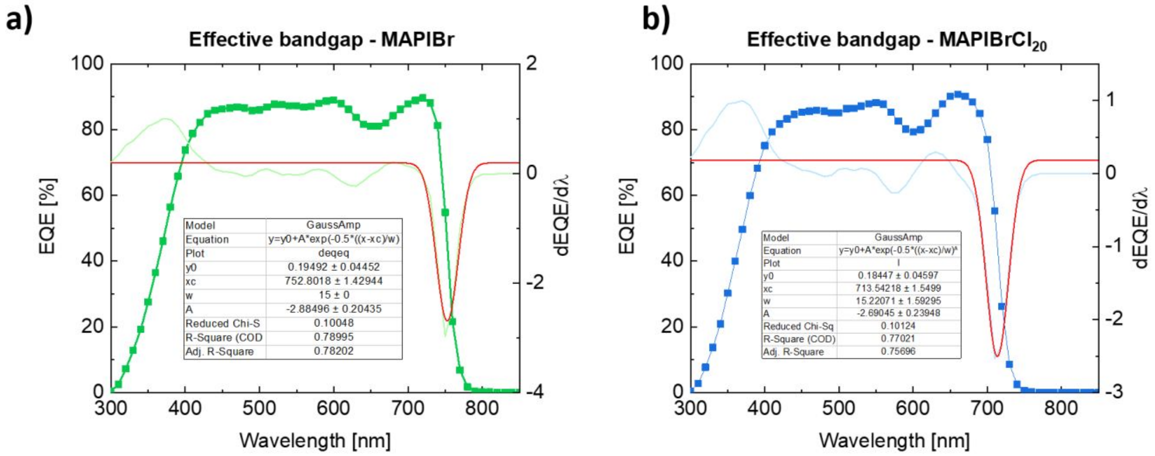

Figure S3. Derivation of the effective bandgap of the PSCs presented in **Figure 4b**.

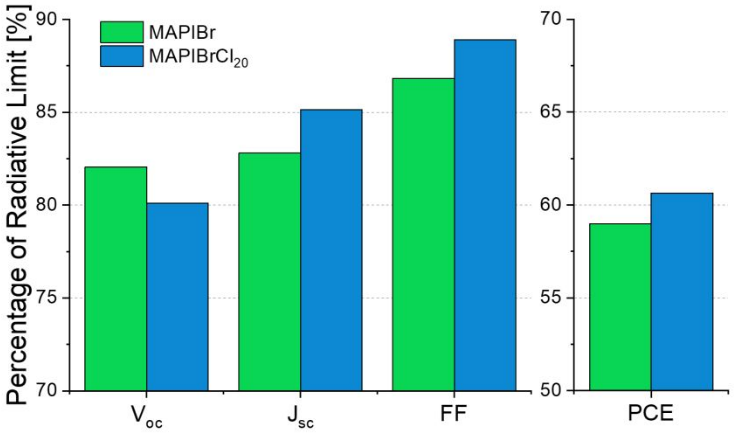

Figure S4. Percentage of the average JV parameters from **Figure 4d** with respect to their radiative limit for MAPIBr and $MAPIBrCl_{20}$. Values of the radiative limit were taken from the literature[5] for a 1.65 and 1.74 eV bandgap respectively, as derived from their respective EQE. The $MAPIBrCl_{20}$ devices could reach more than 60% of the theoretical maximum efficiency, higher than the MAPIBr reference.

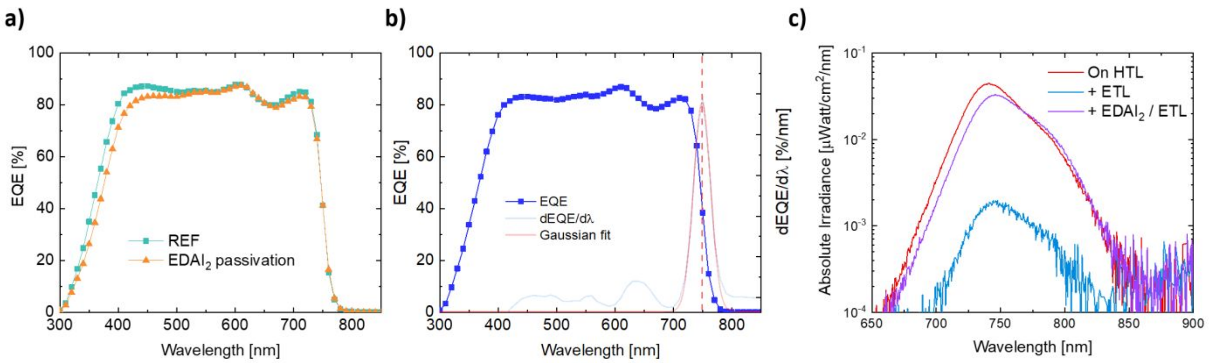

Figure S5. a) EQE spectra of a $MAPIBrCl_{20}$ device with and without EDAI passivation. b) Determination of the effective bandgap through derivation of that same EQE, giving a value of 1.66 eV. c) PL signal of the perovskite film on top of the Hole Transport Layer (HTL), with Electron Transport Layer (ETL) or with $EDAI_2$ + ETL deposited on top, showcasing the surface passivation effect of $EDAI_2$.

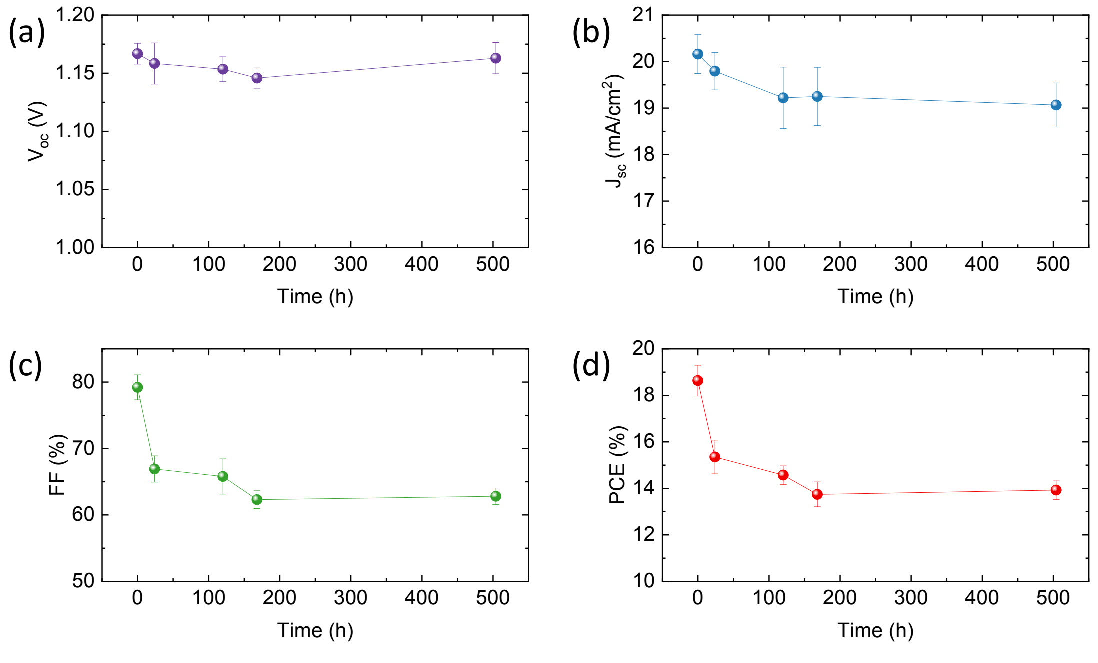

Figure S6. Evolution of the PV parameters obtained from JV scans, for unencapsulated solar cells based on $MAPIBrCl_{20}$ stressed for 500 hours at 85 °C in the dark and nitrogen atmosphere.

References

(1) Ventosinos, F.; Koffman-Frischknecht, A.; Herrera, W.; Senno, M.; Caram, J.; Perez, M. D.; Schmidt, J. A. Estimation of Carrier Mobilities and Recombination Lifetime in Halide Perovskites Films Using the Moving Grating Technique. *J. Phys. Appl. Phys.* **2020**, *53*, 415107. https://doi.org/10.1088/1361-6463/ab9d59.

(2) Haken, U.; Hundhausen, M.; Ley, L. Moving Grating Technique: A New Method for the Determination of Electron and Hole Mobilities and Their Lifetime. *Appl. Phys. Lett.* **1993**, *63*, 3066–3068. https://doi.org/10.1063/1.110260.

(3) FathAllah, A.; Ventosinos, F.; Longeaud, C. An Automated Experiment for Determination of Thin Film Semiconductor Transport Parameters. *J. Phys. Conf. Ser.* **2014**, *558*, 012011. https://doi.org/10.1088/1742-6596/558/1/012011.

(4) Ritter, D.; Zeldov, E.; Weiser, K. Steady-state Photocarrier Grating Technique for Diffusion Length Measurement in Photoconductive Insulators. *Appl. Phys. Lett.* **1986**, *49*, 791–793. https://doi.org/10.1063/1.97548.

(5) Rühle, S. Tabulated Values of the Shockley–Queisser Limit for Single Junction Solar Cells. *Sol. Energy* **2016**, *130*, 139–147. https://doi.org/10.1016/j.solener.2016.02.015.
